# Identification of Candidate Genes for Pigmentation in Camels Using Genotyping-by-Sequencing

**DOI:** 10.3390/ani12091095

**Published:** 2022-04-23

**Authors:** Morteza Bitaraf Sani, Javad Zare Harofte, Mohammad Hossein Banabazi, Asim Faraz, Saeid Esmaeilkhanian, Ali Shafei Naderi, Nader Salim, Abbas Teimoori, Ahmad Bitaraf, Mohammad Zadehrahmani, Pamela Anna Burger, Nader Asadzadeh, Mohammad Silawi, Afsaneh Taghipour Sheshdeh, Behrouz Mohammad Nazari, Mohammad Ali Faghihi, Zahra Roudbari

**Affiliations:** 1 Animal Science Research Department, Yazd Agricultural and Natural Resources Research and Education Center, Agricultural Research, Education & Extension Organization (AREEO), Yazd 8915813155, Iran; javadzare49@gmail.com (J.Z.H.); ashnaderi@gmail.com (A.S.N.); a_btrf@yahoo.com (A.B.); 2 Animal Science Research Institute of Iran, Agricultural Research, Education and Extension Organization (AREEO), Karaj 3146618361, Iran; hossein.banabazi@gmail.com (M.H.B.); esmaeilkhanian@yahoo.com (S.E.); naderasadzadeh4@gmail.com (N.A.); 3 Department of Animal Breeding and Genetics (HGEN), Centre for Veterinary Medicine and Animal Science (VHC), Swedish University of Agricultural Sciences (SLU), 75007 Uppsala, Sweden; 4 Department of Livestock and Poultry Production, Bahauddin Zakariya University, Multan 60000, Pakistan; drasimfaraz@bzu.edu.pk; 5 Organization of Agriculture—Jahad-Yazd, Ministry of Agriculture-Jahad, Yazd 8915813155, Iran; nadersalim390@gmail.com (N.S.); teimoori.abbas@yahoo.com (A.T.); 6 Yazd Dar Al-Elm Higher Education Institute, Yazd 8915813155, Iran; mbetaraf58@gmail.com; 7 Research Institute of Wildlife Ecology, Vetmeduni Vienna, 1160 Vienna, Austria; pamela.burger@vetmeduni.ac.at; 8 Persian Bayan Gene Research and Training Center, Shiraz 7134767617, Iran; silawimohammad@yahoo.com (M.S.); afsane_t1989@yahoo.com (A.T.S.); mohammadalifaghihi2@gmail.com (M.A.F.); 9 Animal Breeding Canter of Iran, Karaj 3173945591, Iran; bmnazari@gmail.com; 10 Department of Animal Science, Faculty of Agriculture, University of Jiroft, Jiroft 7867155311, Iran; rodbari.zahra@gmail.com

**Keywords:** dromedary, pigmentation, coat color, genotyping-by-sequencing

## Abstract

**Simple Summary:**

The coat color of dromedary is usually uniform and varies from black to white. We identified 9 significant SNPs associated with white color, and the 13 significant SNPs associated with black color using genotyping-by-sequencing (GBS). Among candidate genes, SNAI1 that interacts with MCIR, ASIP and KIT genes plays a key role in the melanin biosynthetic and pigmentation biological process and melanogenesis biological pathway.

**Abstract:**

The coat color of dromedary is usually uniform and varies from black to white, although dark- to light-brown colors are the most common phenotypes. This project was designed to gain knowledge on novel color-related variants using genotyping-by-sequencing (GBS). The association between the SNPs and coat color was tested using MLM (mixed linear models) with kinship matrix. Three GWAS models including white color vs. non-white color, black vs. non-black color, and light-brown vs. dark-brown color were performed. There were no distinct genetic clusters detected based on the color phenotypes. However, admixture occurred among all individuals of the four different coat color groups. We identified nine significant SNPs associated with white color after Bonferroni correction, located close to *ANKRD26, GNB1, TSPYL4, TEKT5, DEXI, CIITA, TVP23B, CLEC16A, TMPRSS13, FXYD6, MPZL3, ANKRD26, HFM1, CDC7, TGFBR3*, and *HACE1* genes in neighboring flanking regions. The 13 significant SNPs associated with black color and the candidate genes were: *CAPN7, CHRM4, CIITA, CLEC16A, COL4A4, COL6A6, CREB3L1, DEXI, DGKZ, DGKZ, EAF1, HDLBP, INPP5F, MCMBP, MDK, SEC23IP, SNAI1, TBX15, TEKT5, TMEM189, trpS, TSPYL4, TVP23B,* and *UBE2V1*. The SNAI1 gene interacted with MCIR, ASIP and KIT genes. These genes play a key role in the melanin biosynthetic and pigmentation biological process and melanogenesis biological pathway. Further research using a larger sample size and pedigree data will allow confirmation of associated SNPs and the identified candidate genes.

## 1. Introduction

There is a large diversity of coat colors and patterns in livestock, which is important for an easy and rapid discrimination among breeds, species, or ecotypes [1]. Often this trait has been artificially selected, as the value of natural-colored wool depends on the fiber quality and coat color of the animal (e.g., merino sheep, cashmere goat, alpaca) [2]. Additionally, selective breeding in farm animals has been traditionally based on morphological characters based on coat coloration [3]. However, natural selection for adaptation to environment also acts on coat color variation [4], e.g., black-coated animals attract heat more than others in harsh deserts, supporting resistance to high thermal differences [5]. The color of dromedaries is uniform and varies from white (e.g., Wodh) to black (e.g., Magaheem). While few West African camels are spotted [6], dark- to light-brown colors are the most common phenotypes. Based on these colors, Saudi Arabian camels were divided into four breeds: white (Magateer), brown (Al Homr and Al Sofr), and black (Magaheem) [7]. Heredity of coat color is a favorite area of genetics research because of its highly visible nature [8]. The biogenesis of pigment granules is affected by multiple genes [9], and more than 50 genes have been reported affecting coat color in mammals [10]. Well-defined genes for influencing coat color are the agouti signaling protein (ASIP or Agouti), melanocyte-stimulating hormone receptor (MC1R), and proto-oncogene receptor tyrosine kinase (KIT) [11,12,13,14,15,16,17,18,19,20,21,22]. *MC1R* and *ASIP* affect the ratio of eumelanin and pheomelanin distribution in mammals and particularly in domesticated animals [1,3,17]. Eumelanin is responsible for black, brown, or grey and pheomelanin for red, yellow or cream color, respectively [23]. In camelids, a few projects have already studied coat color [1,6,14,24,25,26]. In alpaca, it has shown that *MC1R* and *ASIP* genes are the main candidates for coat color [25]. Three SNPs located on MC1R (901 (C/T)), and TYRP1 (113 (C/T) and 200 (C/T)) were identified in Pakistani dromedaries [24]. ASIP and MCIR were recognized as candidate genes for black and white coat colors, respectively, in dromedaries [1]. The protein *MC1R* plays a key role in pigmentation and determines coat color via dark eumelanin or light pheomelanin [1]. α-MSH as a melanocyte stimulating hormone connected to the *MC1R* receptor to produce eumelanin. It seems that *ASIP* gene regulates expression of *MC1R* gene and reduces eumelanin, causing the brown coat color in dromedary [1]. Although most dromedary populations are brown, its intensity differs from white to black color in different breeds and populations of dromedaries, which can be due to epistatic interactions with other gene products besides those from *MC1R* and *ASIP* genes [1]. With next generation sequencing platforms and genome-wide association studies it is becoming more and more efficient to study the mechanism of pigmentation. In Iran, there are about 140,000 dromedaries [27]. They are usually light- and dark-brown color. 

We designed this project to gain knowledge on novel color-related variants, and we applied a genome-wide association study approach using genotyping-by-sequencing (GBS). We aimed at identifying novel SNPs associated with pigmentation in dromedaries. The ultimate goal of the study was to identify proteins associated with the various shades of pigmentation observed in this livestock species. 

## 2. Materials and Methods

The work was approved by ASRI’s Animal Ethics Committee (the number ASRI-34–64-1357–005-970,180). The blood samples were gathered from 96 dromedaries in Iran central desert including the following: light-brown (*n* = 42), dark-brown (*n* = 35), black (*n* = 9), and white (*n* = 10). DNA Extraction was performed by the modified salt precipitation method [28]; after elution, quality was checked by spectrophotometry. Finally, DNA samples were adjusted to a concentration of 50 ng/µL for subsequent steps. EcoR1 and HinF1 enzymes were used to genotyping-by-sequencing, based on paired-end (150 bp) sequencing. GBS library was composed in three steps: cutting the DNA using Restriction enzymes, attaching adaptor to the cut DNA, and amplifying DNA Molecules using DNA polymerase. This project was carried out using the Illumina HiSeq 2000 platform in Persian Bayangene Research and Training Center (Shiraz, Iran). Trimming of adapters and quality control of read pairs (base Qphred ≤20) were performed using bcl2fastq V2.20 and fastQC V1.0.0 tools. Mapping of sequence reads (GCA_000803125.3) [29], PCR duplicates detection, and SNP calling were executed using the BWA-MEM algorithm of Burrows–Wheeler Aligner (BWA) [30], Picard tools [31], SAMtools [32], and GATK, respectively. Variants with MAF <0.05 and Call Rate < 0.95 were removed. The association between the SNPs and coat color were estimated by MLM (mixed linear models) with kinship matrix [32]. In this project, the regions of sampling were included as fixed effect. We performed the case–control GWAS in three sets. In set I, we treated all the white colors as case groups (*n* = 10) and non-white appearance as controls (*n* = 86). In set II, the black colors were treated as case groups (*n* = 9) and non-black appearance as controls (*n* = 87). In the remaining set, we treated light brown (*n* = 42) as case groups and dark brown color as controls (*n* = 35). 

The statistical model was as follows:

Y = αX + UZ + e

Y: coat color
α: SNP effects
U: kinship background effects
e: residual effects
X and Z: incidence matrix relating the individuals to fixed marker effects α and random group effects u, respectively.

We used Bonferroni-corrected threshold (-log *p* value > 4.16), which were defined as 0.05/N (N is the number of tested SNPs). The candidate genes were detected by tracing the associated SNPs in NCBI (GCA_000803125.3) [29], located either within the exon/intron of a gene or within a flanking region of 100 kb up- and downstream. vcfR, poppr, ape, and RColorBrewer packages in R V4.1.2 [33,34,35,36] were used for K-means clustering and discriminant analysis of principal components (DAPC) and TASSEL V5.0 [37] for generation of Manhattan and q–q plot. PPI enrichment analysis was performed by STRING database [38].

## 3. Results

### 3.1. Summary Statistic of SNPs, Linkage Disequilibrium, Population Structure, and Kinship Analyses

**Figure 1:**
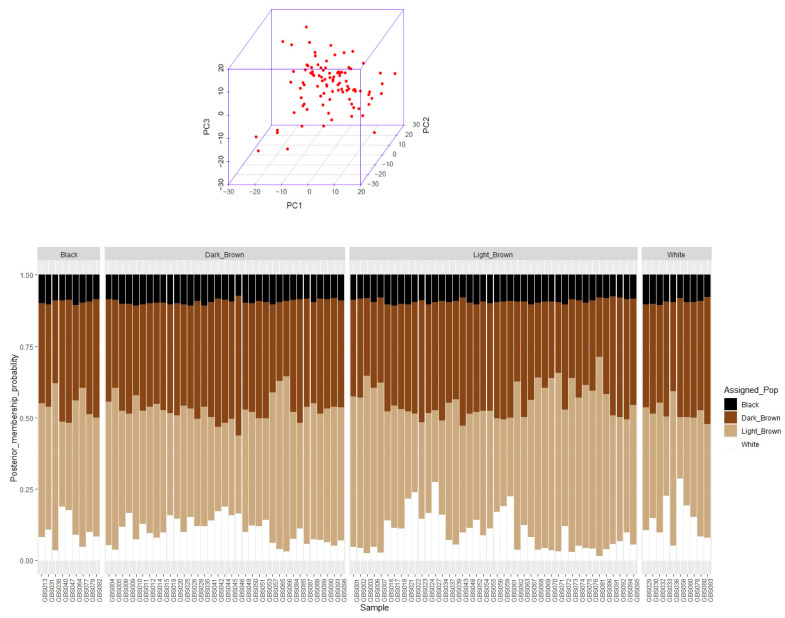
Principal component analysis of 96 dromedary camels of Iran central desert using 14,522 SNPs in five regions in above figure (**A**). Admixture of 96 dromedary camels among four coat colors: light-brown, dark-brown, black, and white, where the x-axis shows sample number and the y-axis show the probability of each camel belongs to coat color (color = K) in bottom figure (**B**).

The number of 41,897 variants were discovered. A total of 14,522 SNPs remained after quality control, so that 256 monomorphic markers and 27,375 markers with MAF < 0.05 were removed. Principle component analysis (PCA) showed that the camels of five different regions of Iran central desert were homogenous (Figure 1A). As there were no distinctive clusters detected based on sampling regions, admixture among all individuals of the four different coat colors must have occurred (Figure 1B). 

### 3.2. Genome-Wide Association Study

**Figure 2:**
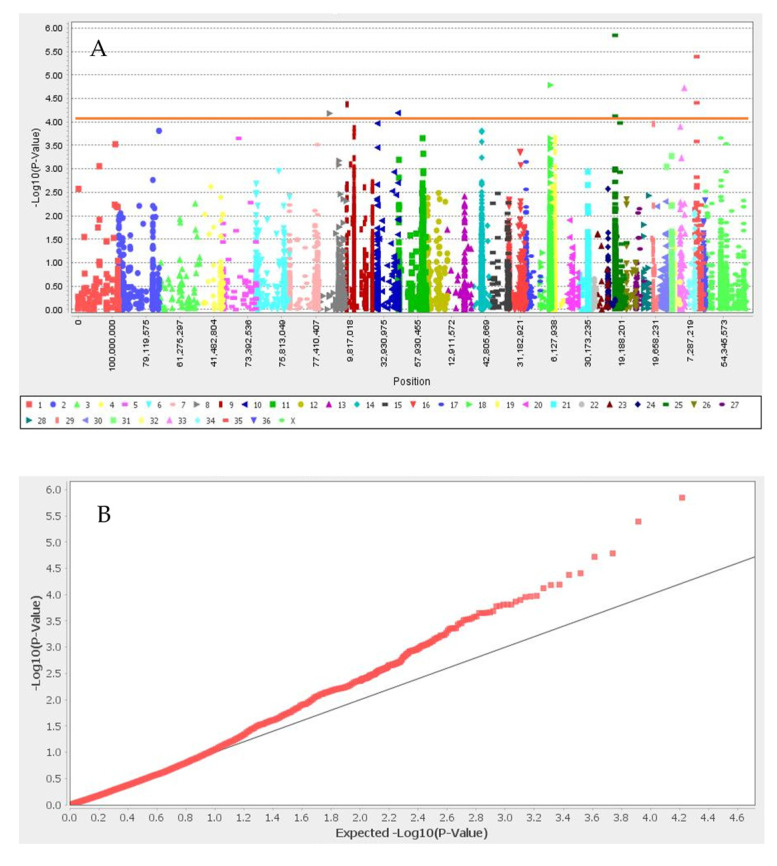
Manhattan plot (**A**) and q–q plot (**B**) of coat color for white color. Red line in Manhattan plot is Bonferroni threshold. The chromosomal position of each SNP was displayed in different colors in Manhattan plot. Red and gray line in the q–q plots represent the −log *p*-value of the entire study and expected value, respectively.

**Figure 3:**
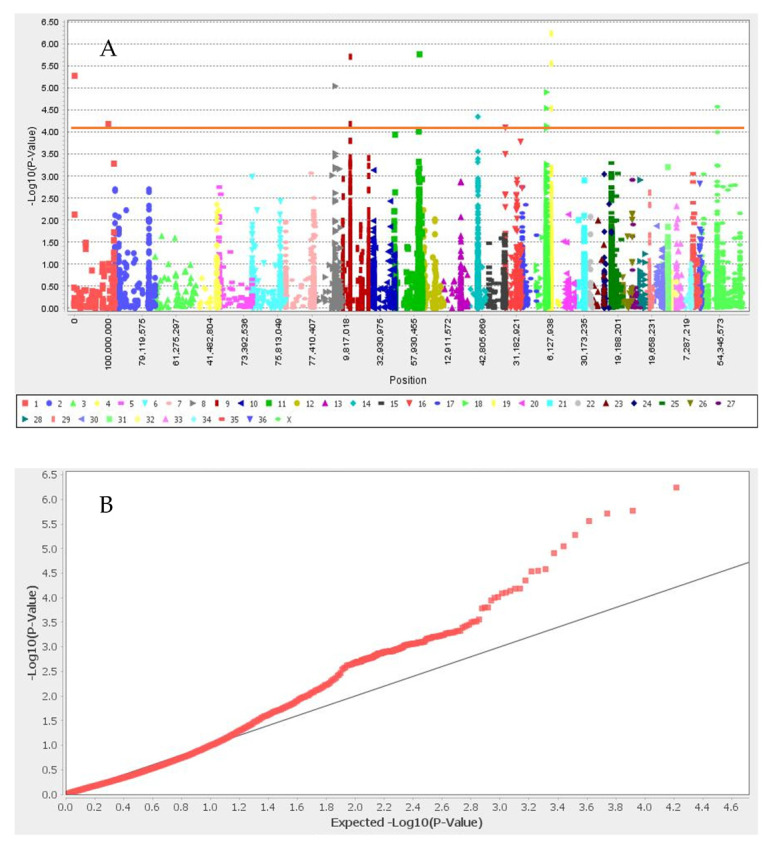
Manhattan plot (**A**) and q–q plot (**B**) of coat color for black color. Red line in Manhattan plot is Bonferroni threshold. The chromosomal position of each SNP was displayed in different colors in Manhattan plot. Red and gray line in the q–q plots represent the −log *p*-value of the entire study and expected value, respectively.

GWAS results for the set I and set II are presented in Figure 2 and Figure 3, respectively. We identified nine significant SNPs associated with white color after Bonferroni correction, located in neighboring flanking regions close to *ANKRD26, GNB1, TSPYL4, TEKT5, DEXI, CIITA, TVP23B, CLEC16A, TMPRSS13, FXYD6, MPZL3, HFM1, CDC7, TGFBR3,* and *HACE1* genes in neighboring flanking regions (Table 1 and Figure 2). The 13 significant SNPs associated with black color and their candidate genes were: *CAPN7, CHRM4, CIITA, CLEC16A, COL4A4, COL6A6, CREB3L1, DEXI, DGKZ, DGKZ, EAF1, HDLBP, INPP5F, MCMBP, MDK, SEC23IP, SNAI1, TBX15, TEKT5, TMEM189, trpS, TSPYL4, TVP23B,* and *UBE2V1*. We did not find any SNPs associated with light- and dark-brown color in this study (Table 1 and Figure 3). SNP Chr19_10157184 located close to SNAI1 gene that the result of PPI enrichment analysis by using STRING database indicated *SNAI1* gene interacts with *MCIR*, *ASIP* and *KIT* genes. Network analysis of *SNAI1, MCIR, ASIP*, and *KIT* genes showed that the number of nodes is eight, the number of edges is 17, the average degree of node is 4.25, the clustering coefficient is 0.86 and it is significant based on *p*_value < 0.001.

## 4. Discussion

In this research, genome-wide association analysis with 15K SNPs has been per-formed with an attempt to clarify the SNPs associated with various coat colors exhibited in dromedaries. Most dromedaries from Iran central desert included in this study had brown coat colors. We identified 22 markers that were significantly associated with coat color in dromedaries; thus, nine SNPs included the following: Chr25_73462, Chr35_5648539, Chr18_29916170, Chr33_12297059, Chr35_5696438, Chr9_1807083, Chr10_74907708, Chr8_31826040, and Chr25_505194 related with white color; 13 SNPs included the following: Chr19_10157184, Chr11_74286851, Chr9_22746017, Chr19_10612243, Chr1_2037209, Chr8_59919441, Chr18_29898490, Chrx_46816486, Chr18_29898527, Chr19_10612244, Chr14_30853969, Chr9_22614201, and Chr1_101414918 with black color. The marker called Chr35_5648539, associated with white color, located near to *GNB1* gene and functional profiling of this gene, suggested phototransduction with KEGG: 04744 (*p*-value = 2.39 × 10^−2^). The marker called Chr1_2037209, associated with black color, located near to *COL4A4, COL6A6* genes and collagen type IV trimer term with GO:0005587 (*p*-value = 2.90 × 10^−2^), was detected for these genes. The marker called Chr19_10157184, located close to *SNAI1*, interacted with *MCIR*, *ASIP* and *KIT* genes, which together play a key role in the melanin biosynthetic and pigmentation biological process and melanogenesis biological pathway. The black and brown coat colors are probably caused by the *ASIP* gene in dromedaries [1], while the white coat color is likely regulated by the *MC1R* gene in Wodh dromedaries [1]. Heterozygosity of c.901C >T in MC1R as well as the homozygous genotype of *ASIP* exon 2 are responsible for the white color phenotype. Likewise, several researchers have reported *MC1R*, *ASIP* genes related to coat color in cattle, goats, and sheep [39,40,41,42,43,44,45,46]. Holl et al. (2017) found that a mutation in the *KIT* gene is associated with white-spotted phenotypes in the dromedary [22]. *TYR* is another basic gene that regulates pigmentation [47] and disorders in this gene cause the albino phenotype in mammals and chickens [48]. Genetic diversity of indigenous dromedaries is at risk in Iran [49], their genetic diversity needs to be conserved. One of approaches to prediction of animal breed is coat color, so that morphological selection in domestic animals based on color determines the breed and attribution [17]. The reported SNPs in this study represent the candidate polymorphisms associated with pigmentation in dromedaries and can be used in future genetic studies. However, whole genome sequencing, pedigree, and more phenotype data will be required to accurate survey of coat color in dromedaries.

## 5. Conclusions

Fiber color is important in textile industry [50], and the Asian camel farmers show high interest for breeding white and black dromedaries [1]. Genetic analyses indicated that several loci are involved in coat color in dromedaries that can be used in selective breeding or towards identification of purebred animals. It would be very interesting to survey indigenous dromedaries regarding their color evolution, using a larger sample size and pedigree which should include many populations and breeds. In addition, more detailed studies are needed in order to understand the associated SNPs and candidate genes identified in this project. 

## Figures and Tables

**Table 1:** The associated SNPs in three case–control GWAS models of coat color in dromedaries.

Coat Color	SNP	Chr	Pos	−log (*p*-Value)	Candidate Genes ± 100 kb
White	Chr25_73462	25	734,62	5.84	-
	Chr35_5648539	35	5,648,539	5.39	*ANKRD26,* GNB1
	Chr18_29916170	18	29,916,170	4.78	*TSPYL4, TEKT5, DEXI, CIITA, TVP23B, CLEC16A*
	Chr33_12297059	33	12,297,059	4.71	*TMPRSS13, FXYD6, MPZL3, ANKRD26*
	Chr35_5696438	35	5,696,438	4.40	-
	Chr9_1807083	9	1,807,083	4.37	*HFM1, CDC7, TGFBR3*
	Chr10_74907708	10	74,907,708	4.19	-
	Chr8_31826040	8	31,826,040	4.17	*HACE1*
	Chr25_505194	25	505,194	4.16	-
Black	Chr19_10157184	19	10,157,184	6.24	*UBE2V1, TMEM189, SNAI1*
	Chr11_74286851	11	74,286,851	5.77	*SEC23IP, MCMBP* *, INPP5F*
	Chr9_22746017	9	22,746,017	5.71	*trpS*
	Chr19_10612243	19	10,612,243	5.56	*CREB3L1, DGKZ, MDK, CHRM4*
	Chr1_2037209	1	2,037,209	5.28	*COL4A4, COL6A6, CAPN7, EAF1*
	Chr8_59919441	8	59,919,441	5.05	
	Chr18_29898490	18	29,898,490	4.91	*TSPYL4, TEKT5, DEXI, CIITA, TVP23B, CLEC16A*
	Chrx_46816486	X	46,816,486	4.58	*HDLBP*
	Chr18_29898527	18	29,898,527	4.55	*TSPYL4, TEKT5, DEXI, CIITA, TVP23B, CLEC16A*
	Chr19_10612244	19	10,612,244	4.54	*CREB3L1, DGKZ, MDK, CHRM4*
	Chr14_30853969	14	30,853,969	4.35	*-*
	Chr9_22614201	9	22,614,201	4.19	*TBX15, trpS*
	Chr1_101414918	1	1.01 × 10^8^	4.18	*-*
Light and dark brown	No associated SNP				

## Data Availability

The datasets generated and during the current study are available in the [dryad] repository, [https://datadryad.org/stash].(accessed on 22 April 2022).
